# Shoot Induction from Axillary Shoot Tip Explants of Fig (*Ficus carica*) cv. Japanese BTM 6

**DOI:** 10.21315/tlsr2018.29.2.11

**Published:** 2018-07-06

**Authors:** Wan Ting Ling, Fui Chu Liew, Wei Yong Lim, Sreeramanan Subramaniam, Bee Lynn Chew

**Affiliations:** 1 School of Biological Sciences, Universiti Sains Malaysia, 11800 USM Pulau Pinang, Malaysia; 2 Fig Direct Sendirian Berhad, Taman Rajawali Indah, Jalan Langgar, 05460 Alor Setar, Kedah, Malaysia

**Keywords:** Axillary Shoot Tip, Multiple Shoot Induction, *Ficus carica*, 6-Benzylaminopurine, Zeatin, Pucuk Aksilari, Induksi Pucuk, *Ficus carica*, 6-Benzylaminopurine, Zeatin

## Abstract

Fig, or *Ficus carica*, is a fruit tree from the Moraceae family and is widely grown in tropical and subtropical regions of the world. Fig plants are mainly propagated through grafting, air layering, and hardwood cutting whereby these methods were found to be less efficient. Plant tissue culture is efficient method to propagate plants, particularly to produce true-to-type platelets for mass multiplication. The aim of this study is to induce multiple shoot formation on *Ficus carica* cv. Japanese BTM 6 through identifying and optimising the concentrations of 6-Benzylaminopurine (BAP) and Zeatin suited for shoot formation. The axillary shoot tip explants were cultured in MS media supplemented with different concentrations of BAP and Zeatin (0, 0.5, 1.0, 1.5 and 2.0 mg/L) to determine the optimal concentration for the formation of multiple shoots. Number of shoots per explants and the differences in shoot height of explants were calculated after 8 and 12 weeks of culture respectively. Of all the treatments of BAP, MS media containing with 2 mg/L BAP marked the highest number of shoots per explant with the average value of 1.67 ± 0.33 while 1.5 and 2 mg/L of BAP produced the highest differences in shoot height with 0.51 ± 0.08 cm and 0.51 ± 0.07 cm after 12 weeks respectively. Murashige and Skoog (MS) media supplemented with 2 mg/L Zeatin showed the highest production of multiple shoots and differences in shoot height with the average of 0.83 ± 0.219 and 0.32 ± 0.04 cm respectively among all the different treatments of Zeatin. In this study, BAP performed better in shoot induction and elongation as compared to Zeatin for the cultivar Japanese BTM 6.

## Abstract (ms)

Pokok Tin, atau nama saintifiknya *Ficus carica* adalah pokok berbuah yang tergolong dalam famili Moraceae dan didapati di kawasan tropika dan subtropika dunia. Pokok Tin boleh dibiakkan dalam pelbagai teknik seperti cantuman, lenturan udara dan keratan kayu keras. Namun, kaedah-kaedah ini didapati kurang efisien. Kultur tisu tumbuhan merupakan satu kaedah yang cekap dalam pembiakan tumbuh-tumbuhan terutamanya dalam menghasilkan plantlet *true-to-type*. Tujuan kajian ini adalah untuk meningkatkan penggandaan pucuk *Ficus carica* dengan mengenal pasti dan mengoptimumkan penggunaan hormon 6-Benzylaminopurine (BAP) dan Zeatin yang sesuai untuk pertumbuhan pucuk. Eksplan pucuk aksilari telah dikultur secara berasingan pada kepekatan BAP dan Zeatin yang berbeza (0.0, 0.5, 1.0, 1.5 dan 2.0 mg/L) untuk menentukan kepekatan optimum bagi penggandaan pucuk. Penggandaan pucuk yang terhasil daripada pucuk eksplan dan purata panjang pucuk eksplan direkod selepas 8 dan 12 minggu. Antara semua rawatan dengan BAP, MS media yang mengandungi 2 mg/L BAP menghasilkan pucuk yang tertinggi dengan purata 1.67 ± 0.33 sementara 1.5 dan 2 mg/L BAP menghasilkan purata pucuk yang terpanjang dengan 0.51 ± 0.08 dan 0.51 ± 0.07. Rawatan 2 mg/L Zeatin menunjukkan penghasilan pucuk berganda yang paling banyak dan panjang di antara semua kepekatan Zeatin dengan purata 0.83 ± 0.219 dan 0.32 ± 0.04 cm serta merupakan rawatan yang terbaik untuk ketinggian tumbuhan. Kajian ini telah menunjukkan bahawa BAP menghasilkan penggandaan pucuk dan perbezaan ketinggian tumbuhan yang lebih baik daripada Zeatin untuk kultivar Japanese BTM 6.

## INTRODUCTION

The fig or *Ficus carica* is a fruiting tree of the *Ficus* genus from the family of Moraceae. It is one of the earliest cultivated plant and is distributed throughout various regions including Mediterranean countries, Indian subcontinent, Far East, Latin America, and Southern California (Lansky & Paavilainen 2011). The fig fruit plays a vital symbol in the holy books of the Bible and the Quran and Hadith (Slavin 2006; Marwat *et al.* 2009). The fig fruit is commonly consumed in its dry and fresh form whereby the fruit itself contains high nutritional values that are important for the maintenance and promotion of good health. Fig fruits are rich in fiber, potassium, calcium and iron that are much higher than in bananas, grapes, strawberries, apple and oranges. Dietary fiber and polyphenols are present at high amounts for both dried and fresh figs (Vinson 1999). In addition, the entire fig tree including the fruits, leaves, roots, latex and leafy branches have been utilised ailments for many types of illness which includes eye vision problems, colic treatment, indigestion and diarrhea (Barolo *et al.* 2014). Previous study by Barolo *et al.* (2014) reported the medicinal characteristics of the fig plant which included antioxidant properties, anti-helminthic, anti-fungal, and anti-carcinogenic, hence making it applicable in studies of ethno pharmacology.

At current, fresh figs are being imported at a high price and are not commercially grown in Malaysia for the local consumption. Propagation methods such as cuttings, grafting and air layer growing are being applied for multiplication as seedlings are not preferred due to characteristic differences diverting from mother plants. These existing vegetative methods are seen quicker and more reliable in propagating fig plants of different varieties. However, these methods do not yield consistent growth of plants and this effect the inconsistent production of fruits altogether especially at the commercial level for a larger scale market.

Tissue culture technology such as micropropagation is a reliable and efficient alternative in mass propagating plants *in-vitro* at a consistent and faster rate producing quality clones of novel cultivars. Micropropagation is a more advantageous method compared to conventional methods as plants can be rapidly multiplied without interruption of external factors such as changes in weather, diseases and low seed viability (Akin-Idowu *et al.* 2009). Previous studies on the micropropagation in different fig cultivars have reported successful shoot regeneration via apical buds, apical shoot buds and lateral shoot buds (Fráguas *et al.* 2004; Kim *et al.* 2007; Taha *et al.* 2013; Danial *et al.* 2014; Darwesh *et al.* 2014; Bayoudh *et al.* 2015; Dhage *et al.* 2015). The current study aims to establish an efficient protocol to for shoot formation of *Ficus carica* cv. Japanese BTM 6 via axillary shoot tip explants for the mass multiplication of fig plantlets.

## MATERIALS AND METHODS

### Plant Materials

Axillary shoot tips of *Ficus carica* cv. Japanese BTM 6 were excised at approximately 2–3 cm from mature mother plants grown and maintained at the School of Biological Sciences, Universiti Sains Malaysia.

### Surface Sterilisation of Explants

The shoot tip explants were brushed gently with tap water containing dishwashing liquid (Sunlight^®^) and 3 drops of Tween-20 followed by rinsing under running tap water for 30 min. The explants were then soaked in 70% ethanol for 10 min and rinsed with sterile distilled water for 5 times followed by treatment with 70% Clorox with gentle agitation for 10 min and rinsed 5 times again with sterile distilled water. The surface sterilised explants were dried on sterile filter paper to remove excess moisture.

### Multiple Shoot Induction

Axillary shoot tip explants were excised into the length of 1 cm prior to culture in full strength MS medium supplemented with 8.0 g/L plant agar, 30 g/L sucrose and different concentrations of BAP (0.5, 1.0, 1.5 and 2.0 mg/L) and Zeatin (0.5, 1.0, 1.5, and 2.0 mg/L). The cultures were placed in the culture room maintained at 25 ± 2°C under continuous white fluorescent tubes with the light intensity 32.5 μE m^−2^ sec^−1^. There were six explant replicates for each treatment.

### Data Collection and Statistical Analysis

The number of shoots, percentage of callus induction and type of callus formed were recorded after 8 weeks of culture, while the average shoot height was recorded after 12 weeks of culture. The data were analysed using one-way analysis of variance (ANOVA) followed by mean comparison using Tukey’s test at *p* ≤ 0.05 by using IBM SPSS statistical analysis (version 22).

## RESULTS AND DISCUSSION

### Effect of BAP on Shoot Tip Explants of *Ficus carica*

**Figure 1:**
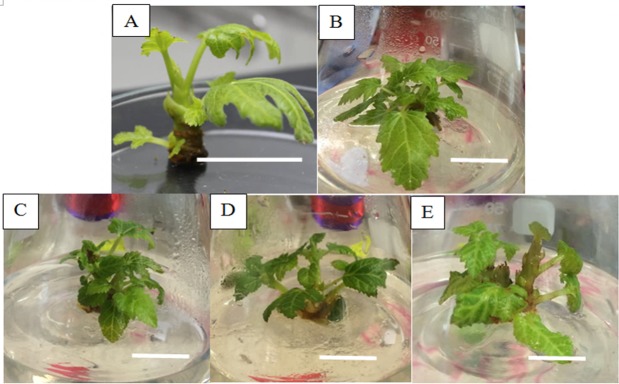
Shoot induction of *Ficus carica* cv. Japanese BTM 6 from axillary shoot tip explants cultured in MS media supplemented with different concentrations of BAP after 8 weeks of culture. A) control; B) 0.5 mg/L BAP; C) 1.0 mg/L BAP; D) 1.5 mg/L BAP; E) 2.0 mg/L BAP. (Scale bar = 1 cm)

With reference to Fig. 1, shoots were induced from axillary shoot tips of *Ficus carica* cv. Japanese BTM 6 cultured in MS media supplemented with 0.5, 1.0, 1.5 and 2.0 mg/L of BAP after 8 weeks of culture. The number of shoots per explant formed for all treatments after 8 weeks of culture is depicted in Table 1. The highest shoot number induced from axillary shoot tip explant was observed in 2.0 mg/L of BAP supplement in MS medium with an average of 1.67 ± 0.33 shoots per explant. This value is significantly higher than the control, 0.5 and 1.5 mg/L BAP treatment.

Danial *et al.* (2014) reported a maximum 5 shoots per explant was successfully induced in MS medium containing 3.0 mg/L BAP via shoot tips of *Ficus carica* L from the Iraqi cultivar. The current study also reported higher concentrations of BAP were able to induce shoots from axillary shoot tips explant whereby higher concentrations of BAP lead to a higher proliferation rate. A study by Darwesh *et al.* (2014) reported the 5.0 mg/L BA and 1.0 mg/L GA (gibberellic acid) supplemented in MS medium induced a significant increment of shoot number in *Ficus carica*. They concluded that benzyladenine (BA) was the most efficient cytokinin in enhancing shoot proliferation of *Ficus carica* L. via shoot tips cultures. In contrary, Mustafa *et al.* (2013) reported on two fig cultivars (Sultany and Aboudi) produced the highest number of induced shoots and leaf from shoot tips explants with the values of 23.79 and 4.29 respectively in full MS media supplemented with as low as 0.5 mg/L BAP. These results differed from the observation in the current study whereby MS media with 0.5 mg/L BAP produced lower response of shoot and leaf induction. These differences in the concentration of BAP used is highly due to the difference in cultivar as variations in the genetic make-up determine the response or interaction between plant growth regulators and endogenous growth regulators of the plant (Verma *et al.* 2009).

In the current study, 100% of callus formation was observed from all treatments of BAP (Table 1). The types of callus formed were semi-friable and yellowish in colour. Calluses are unorganised cell mass formed naturally on plants in response to stress, such as wounding, *Agrobacterium tumefaciens* infection, parasite infection or interspecific hybrids. Fráguas *et al.* (2004) reported that the nodal explants of *Ficus carica* cv. Roxo de Valinhos cultured in the MS medium with BA indicated the formation of callus along with small and vitrified shoots. Danial *et al.* (2014) also discovered that callus formed from the leaf cultures on the media containing the combination of BA with either NAA or Kinetin. They also claimed that the combination of 2,4-D and Kinetin boost the callus induction and weight of *Ficus carica*. A study by Kim *et al.* (2007) discovered the mixture of IBA and TDZ promoted callus induction on leaf segments with more than 80% of shoots regeneration.

The differences in shoot height of all explants were recorded at 12 weeks of culture. With reference to Table 1, there is a significant increase in shoot height within the range of 0.36 cm to 0.51 cm for explants treated with BAP at the concentrations of 1.0, 1.5 and 2.0 mg/L. BAP concentration of 0.5 mg/L did not exhibit significant differences with the control. Darwesh *et al.* (2014) reported on the absence of growth regulator (control treatment) significantly produced the shortest length of shoots while 5.0 mg/L BA combined with 1.0 mg/L GA3 recorded the highest shoot elongation. These results further confirmed the significance of BAP at concentrations above 1.0 mg/L in shoot elongation of *Ficus carica* cv. Japanese BTM 6.

### Effect of Zeatin on Shoot Tip Explants of *Ficus carica*

**Figure 2:**
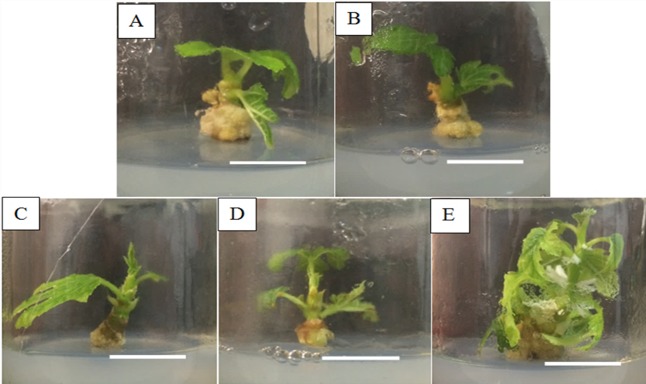
Shoot induction of *Ficus carica* cv. Japanese BTM 6 from axillary shoot tip explants cultured in MS media supplemented with different concentrations of Zeatin after 8 weeks of culture. A) control; B) 0.5 mg/L Zeatin; C) 1.0 mg/L Zeatin; D) 1.5 mg/L Zeatin; E) 2.0 mg/L Zeatin (Scale bar = 1cm)

The axillary shoot tip of *Ficus carica* were cultured on MS medium supplemented with Zeatin with the concentration series of 0.5, 1.0, 1.5, 2.0 mg/L and the observed results is depicted in Fig. 2. With reference to Table 1, no shoots were induced in the MS media without Zeatin, MS media supplemented with 0.5 and 1.0 mg/L of Zeatin. The concentration of 2.0 mg/L of Zeatin indicated shoot formation with a total mean of 0.83 ± 0.219 shoots per explant. Even though MS media containing 1.5 mg/L of Zeatin produced shoots with an average of 0.17 ± 0.09 shoots per explant, this value was found to be insignificant when compared to the control, 0.5, and 1.0 mg/L Zeatin.

Zeatin is a naturally occurring cytokinin obtained from *Zea mays* and is vital in promoting the growth of callus and cell division in callus tissues, as well as inducing shoot formation (Ling *et al*., 2013). Zeatin is commonly used in the micropropagation of varies woody species due to its tremendous ability in stimulating shoot proliferation. Martin *et al.* (2001) discovered that Zeatin is more efficient at inducing cell division than kinetin in carrot roots and soybean callus culture. In this study, Zeatin was used to induce the multiple shoots from *Ficus carica* axillary shoot tips explants and only 1.5 and 2.0 mg/L Zeatin responded with shoot formation.

Similarly, a percentage of 100% callus formation was also observed in all treatments of Zeatin after 8 weeks of culture where callus formed were observed to be semi-friable and yellowish in colour. These observations indicated that Zeatin is indeed more efficient in inducing callus than shoots for *Ficus carica* cv. Japanese BTM 6. These results correlates with the work of Samuelson and Larsson (1993) whereby they reported the efficiency of Zeatin as a cytokinin for callus formation of woody plants.

Based on the results (Table 1), shoot height of the explants were found to be higher for for 2.0 mg/L of Zeatin with the average value of 0.32 ± 0.04 cm. In this study, Zeatin was found to be less effective than BAP in shoot production as the highest concentration used in this study (2.0 mg/L) produced a lower average of 0.83 ± 0.219 shoots per explant. Zeatin is a cytokinin that is commonly used lateral bud formation, stimulation of lateral dominance and induction of multiple shoot from internodes. Cuenca *et al*. (2000) utilised Zeatin together with BA in Woody Plant Medium (WPM) as the proliferation medium for the shoot bud explants of *Fagus sylvatica* and *F.orientalis* indicating the presence other cytokinin may be necessary in proliferation of shoots in *Ficus carica*. On the other hand, previous studies of the micropropagation of figs have reported the use of BAP in successfully inducing multiple shoots indicating that BAP is commonly used for the tissue culture propagation in figs (Kumar *et al.* 1998; Mustafa & Taha 2012; Mustafa *et al.* 2013; Gharbia *et al.* 2014). The differences in shoot height of induced shoots were subsequently increased as Zeatin concentration increased. Rowland and Ogden (1992) reported that WPM medium supplemented with 20 μM Zeatin promoted the regeneration of shoots in leaf explants of highbush blueberry after 13 weeks of culture. Masekesa *et al.* (2016) tested Zeatin, Kinetin, and TDZ on sweet potato cv. Brondal for the induction of shoots and discovered that Zeatin treatment of 0.2 mg/L produced the highest number of shoots among all the kinetin and TDZ treatments. Up to date, Zeatin is not being tested on the shoot proliferation and induction in *Ficus carica*. Hence, the current study is the first to evaluate the efficiency of Zeatin on the cultivar Japanese BTM 6.

## CONCLUSION

MS media containing 2.0 mg/L of BAP recorded the highest number of shoots from *Ficus carica* axillary shoot tip explants. In addition, MS media added with 2.0 mg/L Zeatin induced shoots but the efficiency of Zeatin in promoting multiple shoots in *Ficus carica* cv. Japanese BTM 6 was found to be less efficient in comparison to BAP.

## Figures and Tables

**Table 1:** Effect of different concentrations of BAP and Zeatin (0.0, 0.5, 1.0, 1.5 and 2.0 mg/L) on the number of shoots per explant, percentages of callus induction and type of callus formed after 8 weeks of cultures, and average shoot height after 12 weeks of culture.

Plant growth regulators (PGRs)	Concentration (mg/L)	Number of shoots per explant after 8 weeks of culture	Differences in shoot height after 12 weeks of culture (cm)	Percentage of callus formation after 8 weeks of culture (%)	Type of callus formed after 8 weeks of culture
BAP	0.0	0.0 ± 0.0 ^a^	0.0 ± 0.0 ^a^	100	Semi-friable callus
	0.5	0.17 ± 0.17 ^a^	0.24 ± 0.04 ^ab^	100	Semi-friable callus
	1.0	0.83 ± 0.17 ^ab^	0.36 ± 0.04 ^b^	100	Semi-friable callus
	1.5	0.50 ± 0.34 ^a^	0.51 ± 0.08 ^b^	100	Semi-friable callus
	2.0	1.67 ± 0.33 ^b^	0.51 ± 0.07 ^b^	100	Semi-friable callus

Zeatin	0.0	0.0 ± 0.0 ^x^	0.0 ± 0.0 ^x^	100	Semi-friable callus
	0.5	0.0 ± 0.0 ^x^	0.0 ± 0.0 ^x^	100	Semi-friable callus
	1.0	0.0 ± 0.0 ^x^	0.0 ± 0.0 ^x^	100	Semi-friable callus
	1.5	0.17 ± 0.093 ^x^	0.07 ± 0.05 ^xy^	100	Semi-friable callus
	2.0	0.83 ± 0.219 ^y^	0.32 ± 0.04 ^y^	100	Semi-friable callus

*Note*: Columns with same letter were not significantly different (Tukey’s test, *p* ≤ 0.05).
